# The DNA methylation drift of the atherosclerotic aorta increases with lesion progression

**DOI:** 10.1186/s12920-015-0085-1

**Published:** 2015-02-27

**Authors:** María del Pilar Valencia-Morales, Silvio Zaina, Holger Heyn, F Javier Carmona, Nuray Varol, Sergi Sayols, Enric Condom, José Ramírez-Ruz, Antonio Gomez, Sebastian Moran, Gertrud Lund, Dalia Rodríguez-Ríos, Gladys López-González, Magda Ramírez-Nava, Carmen de la Rocha, Alejandro Sanchez-Flores, Manel Esteller

**Affiliations:** Department of Medical Sciences, Division of Health Sciences, León Campus, University of Guanajuato, 20 de Enero no. 929, 37320 León, Guanajuato Mexico; Cancer Epigenetics and Biology Program (PEBC), Bellvitge Biomedical Research Institute (IDIBELL), Av. Gran Vía s/n km. 2.7, 08907 L’Hospitalet de Llobregat, Barcelona, Catalonia Spain; Department of Pathology, Bellvitge University Hospital, Bellvitge Biomedical Research Institute (IDIBELL), Barcelona, Catalonia Spain; Department of Pathology and Experimental Therapeutics, University of Barcelona, Barcelona, Catalonia Spain; Department of Anatomic Pathology, Hospital Clinic, University of Barcelona, Barcelona, Catalonia Spain; Department of Genetic Engineering, CINVESTAV, Irapuato, Guanajuato Mexico; Bachelor’s Degree in Nutrition Programme, Division of Health Sciences, León Campus, University of Guanajuato, León, Guanajuato Mexico; University DNA Massive Sequencing Unit, Institute of Biotechnology, UNAM, Cuernavaca, Morelos Mexico; Department of Physiological Sciences II, School of Medicine, University of Barcelona, Barcelona, Catalonia Spain; Institució Catalana de Recerca i Estudis Avançats (ICREA), 08010 Barcelona, Catalonia Spain

**Keywords:** Atherosclerosis, Aorta, DNA methylation, Genome-wide analysis

## Abstract

**Background:**

Atherosclerosis severity-independent alterations in DNA methylation, a reversible and highly regulated DNA modification, have been detected in aortic atheromas, thus supporting the hypothesis that epigenetic mechanisms participate in the pathogenesis of atherosclerosis. One yet unaddressed issue is whether the progression of atherosclerosis is associated with an increase in DNA methylation drift in the vascular tissue. The purpose of the study was to identify CpG methylation profiles that vary with the progression of atherosclerosis in the human aorta.

**Methods:**

We interrogated a set of donor-matched atherosclerotic and normal aortic samples ranging from histological grade III to VII, with a high-density (>450,000 CpG sites) DNA methylation microarray.

**Results:**

We detected a correlation between histological grade and intra-pair differential methylation for 1,985 autosomal CpGs, the vast majority of which drifted towards hypermethylation with lesion progression. The identified CpG loci map to genes that are regulated by known critical transcription factors involved in atherosclerosis and participate in inflammatory and immune responses. Functional relevance was corroborated by crossing the DNA methylation profiles with expression data obtained in the same human aorta sample set, by a transcriptome-wide analysis of murine atherosclerotic aortas and from available public databases.

**Conclusions:**

Our work identifies for the first time atherosclerosis progression-specific DNA methylation profiles in the vascular tissue. These findings provide potential novel markers of lesion severity and targets to counteract the progression of the atheroma.

**Electronic supplementary material:**

The online version of this article (doi:10.1186/s12920-015-0085-1) contains supplementary material, which is available to authorized users.

## Background

DNA methylation is an important epigenetic mechanism of transcriptional regulation [1,2]. The DNA methylome undergoes programmed changes during cellular differentiation, but it can be also modified by exogenous stimuli such as the diet and environmental factors, some of which are of the same typology as known atherosclerosis risk factors [3,4]. Therefore, it has been proposed that the latter may act by imposing aberrant, proatherogenic DNA methylation patterns [5,6]. Indeed, differentially methylated sites mapping to genes participating in atherogenesis have been identified by candidate gene-based studies and in a recent epigenome-wide survey of normal and atherosclerotic human aortas, which showed a genome-wide increase in DNA methylation in atherosclerosis [7-9]. Accordingly, biochemical inhibition of DNA methyltransferase activity decreases vascular lesion size in mice [10,11]. Furthermore, epigenome-wide association studies (EWAS) have linked peripheral blood cell DNA methylation profiles of specific loci to hyperlipidemia, hyperglycemia and obesity, thus uncovering candidate circulating epigenetic markers of atherosclerosis and metabolic conditions predisposing to atherosclerosis [12-14].

By exploiting a high-coverage DNA methylation microarray [15] that determines the methylation status of more than 450,000 CpG sites in the human genome, we previously identified a set of CpG loci (dm-CpGs) that are differentially methylated between donor-matched atherosclerotic and normal human aortas [9]. dm-CpG methylation profiles are independent of lesion histological grade in the grade III-VII range, suggesting that they are established relatively early in the evolution of the atheroma. One question left unanswered in our previous study is whether DNA methylation changes at any CpG loci during the progression of aortic atherosclerosis. To address that issue, we searched the original array data [9] for DNA methylation profiles that are significantly correlated with the histological grade of the atheroma.

## Methods

### Human vascular samples

The *post mortem* donor-matched atherosclerotic and non-atherosclerotic portions of human aortas (referred to as “donor-matched sample pairs”) were previously described [9].

Aortic samples were obtained at the Bellvitge Hospital and the Clinic Hospital in Barcelona, Spain according to a protocol approved by the ethics committee of the Bellvitge Hospital (authorization no. PR311/11) and following signed consent by the relatives. *Post-mortem* time was between 3 and 26 h. Atherosclerotic and normal (A and N, respectively) portions were macroscopically identified by a trained Pathology Technician. Approximately 3-cm long segments of each portion were transferred to RNAlater (Ambion) and refrigerated. A fragment was obtained from the middle portion of each segment (either A or N) and the severity of its histological atherosclerosis was scored on the AHA scale by a qualified Pathologist. The remaining samples were transferred to −80°C within 1 day of dissection. Atherosclerotic lesions and the underlying media were used in all the procedures described here. The relevant donor (age, sex) and sample information for the aortic specimens is shown in Additional file 1: Table S1 in Zaina et al. [9].

**Figure 1:**
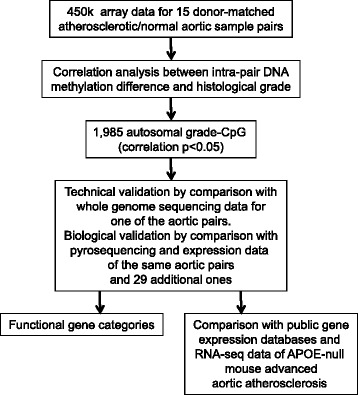
**Sample origin and workflow for the presented data.**

A diagram describing the sample origin and workflow is presented in Figure 1.

### DNA methylation analysis

The microarray-based DNA methylation analysis was conducted with the Infinium HumanMethylation450 BeadChip (450 k array). DNA quality checks, bisulfite modification, hybridization, data normalization, statistical filtering and Beta value calculation were carried out as described elsewhere [15,16]. SNP-harboring probes (11,858) obtained from the list provided by Illumina (support.illumina.com/downloads/infinium_hd_methylation_snp_list.html) were excluded from the analysis. The array data for the samples presented here are extracted from our previously validated and published data set [9], freely available at the Gene Expression Omnibus (GEO) database: http://www.ncbi.nlm.nih.gov/geo/query/acc.cgi?token=zfephwswcyoeotw&acc=GSE46401. The methylation level for each cytosine was expressed as a Beta value calculated as the fluorescence intensity ratio of the methylated to unmethylated versions of the probes. Beta values ranged between 0 (unmethylated) and 1 (methylated). The annotation relating to CGIs uses the following categorization: “shore”, each of the 2 kb-sequences flanking a CGI; “shelf”, each of the 2 kb-sequences next to a shore; “open sea”, DNA not included in any of the previous sequences or in CGIs^2^. TSS200 orTSS1500 indicate the region between position −200 bp or −1,500 bp from the Transcription Start Site (TSS), respectively. Functional gene annotation was performed with the DAVID database (http://david.abcc.ncifcrf.gov). Clustering was performed by Ward’s hierarchical clustering method, using simultaneously samples and CpGs.

Whole genome bisulfite sequencing data of one atherosclerotic (grade VII)/normal human aortic tissue pair were previously published [9]. The complete DNA methylation sequence at the single nucleotide resolution is freely available at the GEO database: http://www.ncbi.nlm.nih.gov/geo/query/acc.cgi?token=zfephwswcyoeotw&acc=GSE46401.

### RNA-seq of mouse aortas

The mouse experimental protocol was approved by the ethics committee of the Department of Medical Sciences, University of Guanajuato, León, Mexico. Apolipoprotein E-null (APOE-null) mice homozygous for the disrupted *Apoe* allele [17] and sex- and age-matched WT siblings were obtained by mating heterozygous mutants that have been maintained in the C57BL/6 background for >100 generations. Mice were fed a normal chow diet (LabDiet 5001) until sacrifice at the age of 1.6 years. After culling by decapitation under anesthesia with Isoflurane (Forane, Baxter), tissues were dissected and kept in RNAlater (Ambion) at −80°C. Aortas were flushed *in situ* with PBS by cardiac puncture after removal of the thoracic and abdominal organs, and carefully cleaned of any fat tissue under the microscope. Trizol-extracted RNA Integrity Number (RIN) and concentration was measured using the Agilent 2100 Bioanalyzer system. After verifying that each sample presented a RIN number > = 7, Illumina RNA-seq libraries were prepared using the TruSeq RNA Sample Preparation Kit, following the vendor instructions and the resulting cDNA libraries were sequenced using the Illumina Genome Analyzer IIx platform with a Paired End configuration of 144 cycles (72 bp read length pairs). The resulting reads were mapped using the BWA aligner (version 0.7.5a-r405) with default parameters [18] to a compilation of CDS sequences from the mouse reference genome (Mus musculus strain C57BL/6 J GRCm38.p2 C57BL/6 J) downloaded from ENSEMBL database using BioMart (http://www.ensembl.org/biomart). Duplicates were discarded by using the Picard MarkDuplicate tool (http://picard.sourceforge.net) and from the filtered alignment, the read count for each gene was calculated. The gene differential expression analysis between the wild type and mutant mouse aortas was performed using the NOIseq R package [19] with TMM normalization and a cutoff line of 0.9 (ranking score) for differential expression significance, which means that a certain gene is about 20 times more likely to be differentially expressed than non-differentially expressed. RNA-seq data are available in the NCBI BioProject database (Project accession PRJNA262445).

### Statistical analysis

In the case of 450 k arrays, Beta values were converted to M values by a Logit transformation [20]. Intra-pair differences in M values were correlated (Spearman’s correlation test) with the histological grade (III-VII range) converted to numeral values. The correlation test was used as a first filter to identify CpGs that exhibit a significant methylation change with histological grade and to determine the direction of that change (i.e. progressive hypermethylation or hypomethylation). The significance of the identified CpGs was corroborated by ANOVA adjusted for age, sex and post mortem time. The significance threshold in whole 450 k array data analysis was a Bonferroni-corrected p < 10^−7^, corresponding to an uncorrected p < 0.05. As for the statistical power, the paired analysis of the same sample (n = 15 pairs) yielded a sizeable number of CpG sites with significant differential methylation after Bonferroni correction [9]. Therefore, we assumed that the sample size would provide the sufficient power for the statistical analysis presented here. As Beta values are a more intuitive representation of methylation profiles, they will be used to describe the data in the Results and Discussion sections. Percentages were compared by using the Chi-square test. The Pearson’s correlation test was used to compare RNA-seq-derived gene expression level with the direction of methylation change (i.e. the correlation r between the delta-M and histological grade). All tests were performed using the STATISTICA software.

## Results

### Aberrant DNA methylation profile of atherosclerotic lesions becomes more frequent with histological grade

We sought DNA methylation profiles that changed with the progression of atherosclerosis. To this end, we conducted a correlation analysis between the methylation M value difference within donor-matched atherosclerotic (A)/normal (N) aortic sample pairs (n = 15) and the respective lesion grade for the >450,000 CpG sites profiled by the Infinium HumanMethylation450 BeadChip (450 k array). Aortic sample histological grade ranged from III to VII. Significantly correlated CpG sites will be referred to as grade-CpGs. We set the following criteria to assign a grade-CpG status: 1) absolute difference >0.05 between the average intra-pair Delta-Betas of the extreme histological grade subsets - i.e. grade III and grade VII. 2) Significant (p < 0.05) Spearman’s correlation rho between histological grade and intra-pair Delta-M, and no significant correlation with donor’s age, sex and post-mortem time. The significant of the correlation test result was further corroborated by submitting the identified CpG set to ANOVA with donor’s age, sex and post-mortem time as covariates (significance threshold p < 0.05). 3) No significant difference between M values of grade VII and grade III N samples, to exclude possible confounding factors that might over- or underestimate the effect of histological grade.

**Figure 2:**
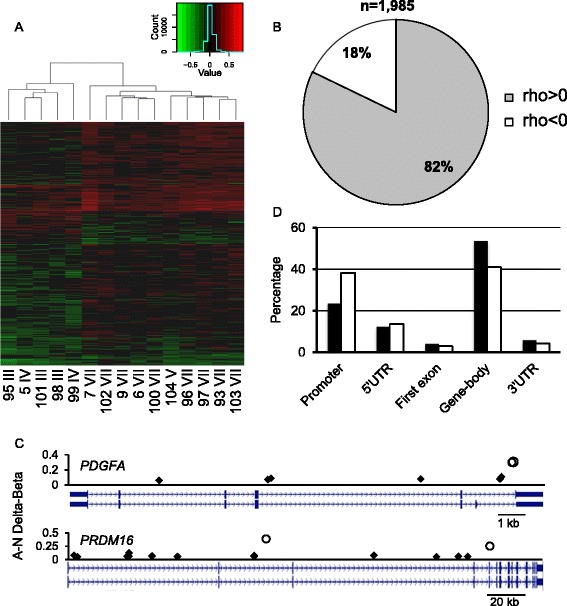
**Features of grade-CpGs. A**, Supervised clustering analysis of the 1,985 grade-CpGs. Donor’s number and corresponding lesion grade are shown for each sample. Note the tendency for hypermethylation in the advanced lesion group. Heatmap color and contrast were enhanced to improve readability, as changes in DNA methylation were overall weak (see intensity distribution above upper right corner of heatmap). **B**, Proportion of grade-CpGs with positive (i.e. hypermethylation with lesion grade) and negative correlation between lesion grade and intra-pair Delta-Beta. **C**, Intra-pair Delta-Beta and position of grade-CpGs (black diamonds) and the previously described dm-CpGs (open circles) in the *PDGFA* and *PRDM16* gene-body. Notice that grade-CpGs reinforce the dm-CpG profiles. **D**, Grade-CpG mapping relative to gene compartments. Solid bars, observed distribution; open bars, distribution of 450 k array probes.

We identified 1,985 autosomal CpG sites (corresponding to 1,206 genes) that matched the grade-CpG criteria (Additional file 1: Table S1). Spearman’s correlation r-values ranged from 0.863 (p = 3.4×10^−5^) to −0.558 (p = 0.031), thus no grade-CpG displayed a significance level below the Bonferroni-corrected p-value. Yet, clustering analysis of the 1,985 grade-CpGs showed a clear grouping by low (III-IV) and high (V-VII) histological grade, with a tendency for hypermethylation in high-histological grade samples (Figure 2A). Accordingly, the vast majority of the grade-CpG methylation profiles (1,631 or 82.2%) correlated positively with atherosclerosis grade, suggesting a gradient change of DNA methylation with disease progression, predominantly towards hypermethylation in advanced lesions (Figure 2B). On average, grade-CpGs displayed weak differential DNA methylation (average absolute grade III versus grade VII A sample Delta-Beta = 0.069; range: 0.050 to 0.522). Notably, 111 genes contained both grade-CpGs and the previously described histological grade-independent dm-CpGs [9]. Thus, grade-CpGs reinforced the DNA methylation drift represented by dm-CpG. As examples, two relevant genes are shown, one involved in vascular smooth muscle cell (VSMC) proliferation (*platelet-derived growth factor alpha polypeptide*, *PDGFA*) [21] and the other participating in the induction of repressive chromatin, in adipose tissue homeostasis and identified as part of the gene expression signature of stable atherosclerotic plaques (*PR domain containing 16*, *PRDM16*) [22-24] (Figure 2C).

As for genomic distribution, grade-CpGs mapped preferentially to gene bodies and were underrepresented in promoters (Chi-square test, p = 0.029, compared with the probe distribution on the 450 k array), whereas their distribution relative to CGIs was only marginally different from expected (p = 0.059) (Figure 2D). When grade-CpGs were grouped according to the direction of methylation change with histological grade, noticeable differences in the genomic distribution were observed. First, CGIs were significantly underrepresented within the hypomethylated grade-CpG set in comparison with the hypermethylated counterpart (6.7% and 21.5%, respectively, Chi-square p = 2.39 × 10^−5^), whereas only a marginal difference in distribution was observed relative to gene compartments (p = 0.07). Second, CpGs undergoing hypomethylation were initially highly methylated - i.e. in the N (baseline) sample, while the hypermethylated counterparts were evenly distributed across the 0–1 Beta range in the N sample (average Beta 0.63 ± 0.20 and 0.48 ± 0.26 for the hypomethylated and hypermethylated grade-CpGs, respectively; *t*-test p = 5.35 × 10^−31^) (Additional file 2: Figure S1A,B). Accordingly, when grade-CpGs of either set were further fractioned by N sample methylation level (high: Beta > 0.75; intermediate: 0.75 ≥ Beta ≥ 0.25; low: Beta < 0.25), a significant underrepresentation of the low methylation fraction within hypomethylated grade-CpGs was observed (7.6% and 26.3% in hypometylated and hypermethylated grade-CpGs, respectively; Chi-square p = 2.3 × 10^−5^) (Additional file 2: Figure S1C). Thus, the histological grade-related hypomethylation and hypermethylation differed, as the former targeted highly methylated CpGs preferentially outside CGIs, in comparison with the latter.

Functionally, grade-CpG-harboring genes were significantly enriched in biological processes involving immune and inflammatory response genes (Fc-gamma receptor-mediated phagocytosis, peroxisome proliferator-activated receptor (PPAR) signaling, T-cell receptor signaling, immune cell-specific expression; Fisher’s exact test, p < 0.05) (Table 1). Noticeably, genes regulated by the transcriptional factors ZEB1 (AREB6) and PPAR-gamma, involved in the cellular response to oxidized lipoproteins and macrophage activation [25,26], respectively, were significantly enriched (949 and 924 genes, p = 4.2 × 10^−10^ and p = 2.4 × 10^−10^) (Table 1). Eighteen grade-CpGs mapped to 9 genes previously identified as differentially methylated in atherosclerosis by candidate gene-based or epigenomics studies (Table 2). Furthermore, 24 grade-CpGs were located within 250 kb from 16 cardiovascular disease-associated single nucleotide polymorphisms (SNPs) [27], suggesting an interplay between the risk genotype and differential DNA methylation as methylation quantitative trait loci (Additional file 3: Table S2) [28]. When grouped according to the direction of methylation change with histological grade, hypermethylated grade-CpG-harboring genes were enriched for the same functional categories as the bulk of grade-CpGs, while no significant enrichment was observed for the hypomethylated counterpart (not shown). Based on our data, we cannot conclude whether the latter result reflects an underlying biological property or is due to a sample size effect.

**Table 1:** **Synopsis of selected relevant biological processes enriched for genes mapping to grade-CpGs (p < 0.05)**

**Category**	**Genes**
Fc-gamma receptor phagocytosis	*DNM3, LYN, PIK3CD, WASF2, PIP5K1B, PIP5K1A, PRKCE, AMPH, MYO10, RAC1, SCIN, PPAP2A, DNM1*
Type 2 diabetes mellitus	*PRKCZ, IRS2, PIK3CD, CACNA1E, MAPK10, MAFA, CACNA1C, PRKCE, CACNA1A, CACNA1B*
T-cell receptor signaling	*HLA-DRB1, PRKAR1B, CREBBP, GNAS, CSK*
Gene regulation by PPAR-alpha	*LPL, PDGFA, PRKAR1B, EHHADH, CREBBP, HSPA1A, NCOR2*
Interaction with ZEB1	949 genes*
Interaction with PPAR-gamma	924 genes*
Tissue specificity:	
Leukocyte	*CAST, KLF6, TRAF2, TNXB, HLA-DRB1, NTF3, MYO7A, RHD, HLA-C, TLR5, MEN1, JUP, CD9, IKBKE, UMPS, ADAP2, NT5E*
Lymphocyte	*HLA-DRB1, HLA-C, HLA-DPB1*

*List available upon request. PPAR, Peroxisome proliferator-activated receptor; ZEB1, Zinc finger E-box binding homeobox 1.

**Table 2:** **Grade-CpG-containing genes identified in previous studies as differentially methylated in atherosclerosis or related metabolic conditions**

**Gene**	**CpG ID**	**Map**	**Correlation rho between Delta-M and lesion grade**
*C1QL4* [29]	cg12760869	Gene-body	0.56
*CTNNA3* [29]	cg04030146	Gene-body	0.54
	cg07002403	Gene-body	−0.53
*DCC* [12]	cg24270629	Gene-body	0.52*
*ESR1* [30]	cg15626350	Gene-body	0.54
*HSPA1A* [31]	cg24888257	First exon	0.53
*IMMT* [29]	cg10973622	Promoter	0.55
*LRP5* [31]	cg22151881	Gene-body	0.53
	cg12016746	Gene-body	0.63
	cg25393429	Gene-body	0.54
	cg14624207	Gene-body	0.57
*PDGFA* [32]	cg06464324	Gene-body	0.59
	cg21473407	Gene-body	0.66
	cg23112425	Gene-body	0.54
	cg19788272	Gene-body	0.59
	cg01130922	Gene-body	0.57
	cg15454385	Gene-body	0.57
*SLC16A3* [33]	cg23664708	3′ UTR	0.52

*Opposite change in DNA methylation in our study and the cited original work.

We performed a technical validation of 450 k data by comparing the promoter grade-CpG methylation profiles with our previous whole genome bisulfite sequencing (WGBS) data for one of the donor-paired aortic samples presented here (sample 93, displaying a grade VII lesion) [9]. Eighteen promoter grade-CpGs mapped to promoter differentially methylated regions (DMRs) identified by WGBS. The correlation between the average intra-pair Delta-Beta of grade VII aortic samples (n = 9) and the intra-pair difference in methylation for the single WGBS aortic sample was significant (r = 0.540, p = 0.021, n = 18), thus ruling out technique-related confounding factors. As for biological validation, the same 450 k array data from which the grade-CpG profiles were obtained, were previously validated in 15 discovery and 24 validation paired aortic samples [9], including the grade-CpG-harboring genes *PDGFA* and *C9orf3/MIR23b*.

### A grade-CpG subset is differentially expressed in human atheromas and in a heterologous model of advanced atherosclerosis

To further corroborate the pathobiological relevance of grade-CpGs, we crossed their methylation profiles with expression data obtained experimentally or from public databases. For the *PDGFA* and *C9orf3/MIR23b* loci, we previously showed that differential methylation coincided with significant changes in expression in aortic samples [9]. Furthermore, 105 grade-CpG-harboring genes overlapped with a human atherosclerotic plaque-specific genes expression signature [24], and 87 overlapped with the list of atherosclerosis-related genes in the HuGENet™ database. The full list of the overlapping genes is shown in Additional file 4: Table S3. Overlapping genes are involved in relevant functions such as lipid homeostasis (*ALOX12*, *CETP*, *LPL*, *LRP1*, *LRP5*, *PLA2G2A*), chemokine signaling (*CCR5*), VSMC proliferation (*PDGFA*), atheroprotection (*ESR1*), local inflammatory response (*VCAM1*, *C3*, *ITGB2*) and T-cell receptor signaling (genes listed in Table 1).

Next, we asked whether any grade-CpG-harboring gene was differentially expressed in a mouse model of advanced atherosclerosis. We reasoned that a strong correlation between human aortic DNA methylation profiles and gene expression in a heterologous model will identify grade-CpGs with potential pathobiological relevance and suitability for comparative mouse model studies. To this end, we performed a RNA-seq-based gene expression analysis in aortic atherosclerotic lesions of one 1.6 years old male APOE-null mouse, a model of hyperlipidemia-induced atherosclerosis [17] and one congenic, sex- and age-matched WT sibling. As expected in a mutant mouse of advanced age, the thoracic aorta showed extensive atheromas (Additional file 2: Figure S2). The relevant descriptive statistics for the RNA-seq analysis of WT and mutant aortic samples is shown in Additional file 5: Table S4. After comparing the samples using the NOIseq R package, among the genes showing a >2-fold expression difference between mutant and control mice, 53 up-regulated and 40 down-regulated genes in the APOE-null mouse harbored grade-CpGs. A total of 99 grade-CpGs mapped to those 93 differentially expressed mouse genes (mgrade-CpGs). The mouse genes and corresponding grade-CpGs are listed in Additional file 6: Table S5. The 23 promoter mgrade-CpGs showed a significant inverse correlation between the histological grade-associated methylation change (i.e. the Spearman’s correlation rho between the methylation status and histological grade) in human aortas and the differential expression of the corresponding mouse genes - i.e. APOE-null relative to WT (Pearson’s r = −0.71, p = 1.5×10^−4^). Thus, the aberrant methylation of promoter grade-CpGs during atherosclerosis progression is associated with an expected expression change of the corresponding gene in a heterologous advanced lesion. A significant inverse correlation for open sea grade-CpG was also observed (r = −0.384, p = 0.009, n = 46), reflecting the abundance of promoter and 5′ gene portions (5′UTR, first exon) mapping to open sea in the mgrade-CpG set (21 out of 46, or 45.6%). The methylation status of gene-body mgrade-CpGs did not show any significant correlation with expression in accordance with the published evidence that intragenic methylation may enhance or repress transcription in a context-specific manner [34,35] nor did CpG island-, shore- or shelf-mapping mgrade-CpGs (data not shown).

As for gene function, 29 of the mgrade-CpG-harboring up-regulated genes in the APOE-null aorta were targets of the master regulator of inflammation nuclear factor kappa B (NFKB) [36], (p = 1.8 × 10^−4^). Interestingly, pathway analysis (BioCarta) revealed enrichment in local inflammatory response and transcription regulation by the arginine methyltransferase CARM1 genes (*PRKAR1B, RARA*) (p = 0.006 and p = 0.05, respectively). These results are consistent with the participation of CARM1 in NFKB transcriptional activation [37]. Of the down-regulated genes, 32 were regulated by myocyte enhancer factor 2A (MEF2A), a transcription factor implicated in coronary artery disease (p = 3.5 × 10^−7^) [38]. Notably, the observation that loss of MEF2A leads to VSMC dedifferentiation is consistent with the enrichment of its targets in the down-regulated gene set [39].

## Discussion

We detected a significant correlation between intra-aortic pair DNA methylation Delta-Beta and lesion histological grade for 1,985 autosomal CpGs (grade-CpGs). Although no grade-CpG lies below the multiple testing-corrected significance level, the results are of potential interest due the convergence of multiple independent supporting evidence. First, we observed a progressive increase in methylation of macrophage function and immunity genes with atherosclerosis progression, reflecting the well-characterized migration of immune and inflammatory cells into the vascular wall and lesion mass during the natural history of atherosclerosis [40]. Second, the hypermethylation of the majority of grade-CpGs with lesion severity represents an exacerbation of the hypermethylated profiles of previously described atherosclerosis-specific dm-CpGs [9]. dm-Cpgs are lesion progression-independent profiles and therefore represent an epigenetic drift of the vascular genome that is already present in low-grade lesions and is amplified by grade-CpGs during lesion progression. Although overlapping in selected loci, the bulk of dm-CpGs and grade-CpGs map to functionally distinct genes, i.e. to SMC-related and immune/inflammatory genes, respectively, pointing to the specific regulation of different pathways in early and progressing atherosclerosis [9]. Noticeably, the hypermethylation of the majority of grade-CpGs in high-grade lesions matches the observation that high expression of the DNA methyltransferase DNMT1 is a signature of inflamed, advanced human plaques [24]. Interestingly, histological grade-related hypermethylation indiscriminately targeted low, intermediate and high methylation CpGs, whereas hypomethylation showed a preference for initially (N sample baseline) highly methylated CpGs in CG-poor regions. Further studies will be necessary to fully elucidate the mechanisms and the biological significance of this observation. Third, the genomic distribution of grade-CpGs generally reflects the one of dm-CpGs, suggesting a common instructive mechanism of DNA methylation drift, particularly the preference for gene-body hypermethylation, despite the functional differences mentioned above. Fourth, several grade-CpGs map to previously identified differentially methylated genes in atherosclerosis. In particular, both a previous epigenomics study carried out in peripheral blood samples [29] and our work in the aorta [9], identify differential methylation in the *C1QL4*, *CTNNA3* and *IMMT* genes. These loci undergo an epigenetic drift towards hypermethylation in independent cardiovascular sample sets representing different tissues and are therefore potential important functional markers. Lastly, we crossed the methylation profiles with available public human databases and transcriptome-wide expression data for a mouse aortic lesion. Although the expression data lack replicates, we used an analysis package (NOIseq) that was developed for differential expression analysis conditions with no replicates as a non-parametric algorithm that evaluates the significance of gene expression changes by comparison against data noise simulation [19]. Hence, the strong inverse association between the methylation status of human promoter grade-CpGs and the expression levels in that heterologous model strongly suggest a general pathobiological relevance for grade-CpGs.

## Conclusion

We provide for the first time a list of CpG loci in the vascular lesion genome that undergo a DNA methylation drift with the progression of the lesion. The identified grade-CpGs represent promising markers of atheroma progression or targets to counteract the progressive DNA hypermethylation associated with the natural history of atherosclerosis.

### Availability of supporting data

The DNA methylation array data have been deposited in the Gene Expression Omnibus (GEO) database: http://www.ncbi.nlm.nih.gov/geo/query/acc.cgi?token=zfephwswcyoeotw&acc=GSE46401.

The RNA-seq data have been deposited in the NCBI BioProject database (www.ncbi.nlm.nih.gov/bioproject) with project accession: PRJNA262445.

## Additional files

Additional file 1: Table S1. List of autosomal grade-CpGs. Additional file 2: Figure S1. Differential distribution of grade-CpGs that undergo hypermethylation or hypomethylation with histological grade, among N sample Beta classes. A,B: N sample Beta of hypermethylated and hypomethylated CpGs (rho>0 and rho<0, respectively). CpGs are ordered for decreasing methylation/grade Spearman’s rho (left to right). C, Distribution of hypermethylated (solid bars) and hypomethylated (open bars) grade-CpGs among high, intermediate and low methylation fractions (left to right). Notice the near-symmetrical distribution of hypermethylated grade-CpGs and the relative underrepresentation of the low methylation fraction among the hypomethylated counterpart. Figure S2: Aortic atherosclerosis in the APOE-null mouse aorta analyzed by RNA-seq. The heart and thoracic aorta of sex-matched, 1.6 years old APOE-null (left) and wt (right) mice are shown. Notice the abundant lesions in the aortic root and arch (arrows) and in the descending aorta in the APOE-null mouse. Additional file 3: Table S2. List of CVD-related SNPs mapping close to grade-CpGs. Additional file 4: Table S3. Grade-CpG-harboring genes overlapping with publicly available CVD gene expression signatures. Additional file 5: Table S4. RNA-seq statistics. Additional file 6: Table S5. Grade-CpG-harboring genes that are differentially expressed in APOE-null and WT mice.
