# Supplementary material for: The DNA methylation drift of the atherosclerotic aorta increases with lesion progression
Source: BMC Med Genomics. 2015 Feb 27;8:7. doi: 10.1186/s12920-015-0085-1 (PMC4353677; doi:10.1186/s12920-015-0085-1)
Supplement: Additional file 2: Figure S1. — Differential distribution of grade-CpGs that undergo hypermethylation or hypomethylation with histological grade, among N sample Beta classes. A,B: N sample Beta of hypermethylated and hypomethylated CpGs (rho>0 and rho<0, respectively). CpGs are ordered for decreasing methylation/grade Spearman’s rho (left to right). C, Distribution of hypermethylated (solid bars) and hypomethylated (open bars) grade-CpGs among high, intermediate and low methylation fractions (left to right). Notice the near-symmetrical distribution of hypermethylated grade-CpGs and the relative underrepresentation of the low methylation fraction among the hypomethylated counterpart. Figure S2: Aortic atherosclerosis in the APOE-null mouse aorta analyzed by RNA-seq. The heart and thoracic aorta of sex-matched, 1.6 years old APOE-null (left) and wt (right) mice are shown. Notice the abundant lesions in the aortic root and arch (arrows) and in the descending aorta in the APOE-null mouse. [file 12920_2015_85_MOESM2_ESM.pdf]

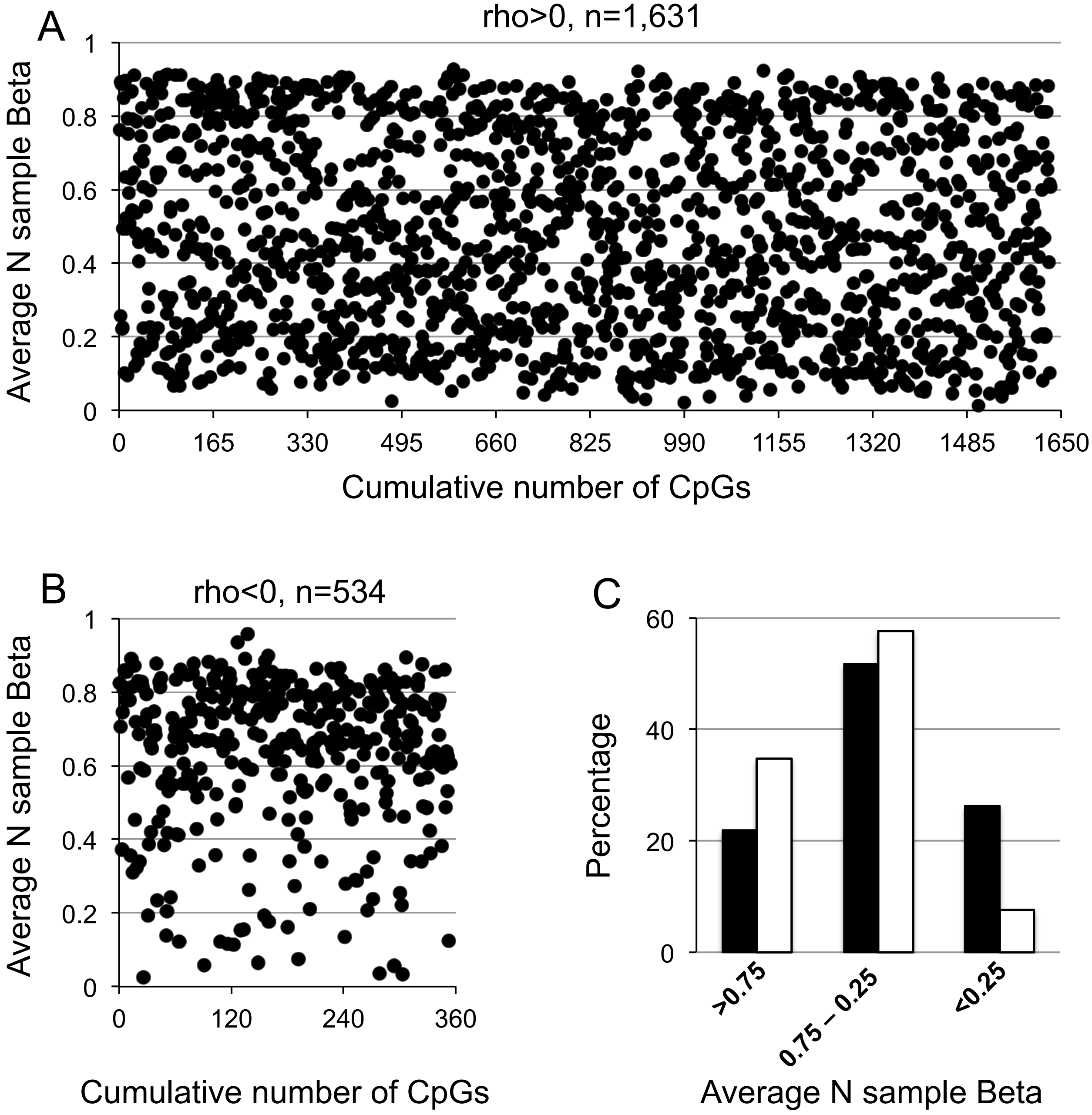

Additional Figure 1. Differential distribution of grade-CpGs that undergo hypermethylation or hypomethylation with histological grade, among N sample Beta classes. A,B: N sample Beta of hypermethylated and hypomethylated CpGs (rho>0 and rho<0, respectively). CpGs are ordered for decreasing methylation/grade Spearman's rho (left to right). C, Distribution of hypermethylated (solid bars) and hypomethylated (open bars) grade-CpGs among high, intermediate and low methylation fractions (left to right). Notice the near-symmetrical distribution of hypermethylated grade-CpGs and the relative underrepresentation of the low methylation fraction among the hypomethylated counterpart.

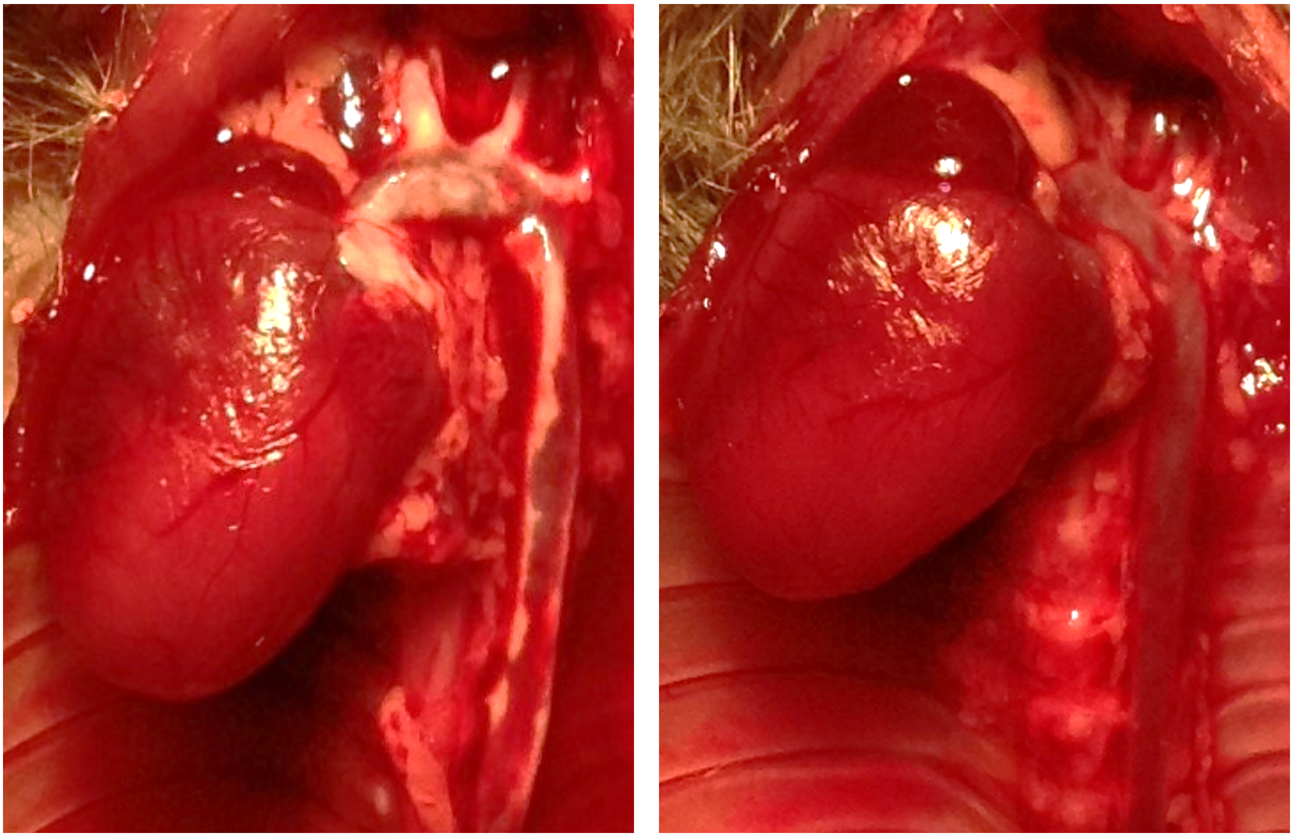

Additional Figure 2. Aortic atherosclerosis in the APOE-null mouse aorta analyzed by RNA-seq. The heart and thoracic aorta of sex-matched, 1.6 years old APOE-null (left) and wt (right) mice are shown. Notice the abundant lesions in the aortic root and arch (arrows) and in the descending aorta in the APOE-null mouse.
