# Supplementary material for: The DNA methylation drift of the atherosclerotic aorta increases with lesion progression
Source: BMC Med Genomics. 2015 Feb 27;8:7. doi: 10.1186/s12920-015-0085-1 (PMC4353677; doi:10.1186/s12920-015-0085-1)
Supplement: Additional file 5: Table S4. — RNA-seq statistics. [file 12920_2015_85_MOESM5_ESM.docx]

**Additional Table 4. RNA-seq statistics.**

______________________________________________

APOE-null aorta WT aorta

______________________________________________

Total reads 18,528,778 16,914,812

Mapped reads 15,787,779 14,596,333

Duplicates 3,803,573 2,540,870

______________________________________________
